# Noradrenaline Improves Behavioral Contrast Sensitivity via the β-Adrenergic Receptor

**DOI:** 10.1371/journal.pone.0168455

**Published:** 2016-12-16

**Authors:** Ryo Mizuyama, Shogo Soma, Naofumi Suemastu, Satoshi Shimegi

**Affiliations:** 1 Laboratory of Cognitive and Behavioral Neuroscience, Graduate School of Frontier Bioscience, Osaka University, Suita, Osaka, Japan; 2 Laboratory of Cognitive and Behavioral Neuroscience, Graduate School of Medicine, Osaka University, Toyonaka, Osaka, Japan; Universidad de Salamanca, SPAIN

## Abstract

Noradrenaline (NA) is released from the locus coeruleus in the brainstem to almost the whole brain depending on the physiological state or behavioral context. NA modulates various brain functions including vision, but many questions about the functional role of its effects and mechanisms remain unclear. To explore these matters, we focused on three questions, 1) whether NA improves detectability of a behavior-relevant visual stimulus, 2) which receptor subtypes contribute to the NA effects, and 3) whether the NA effects are specific for visual features such as spatial frequency (SF). We measured contrast sensitivity in rats by a two-alternative forced choice visual detection task and tested the effects of NA receptor blockers in three SF conditions. Propranolol, a β-adrenergic receptor inhibitor, significantly decreased contrast sensitivity, but neither prazosin nor idazoxan, α_1-_ and α_2-_adrenergic receptor inhibitors, respectively, had an effect. This β blocker effect was observed only at optimal SF. These results indicate that endogenous NA enhances visual detectability depending on stimulus spatial properties via mainly β-adrenergic receptors.

## Introduction

Noradrenaline (NA) is secreted from noradrenergic neurons in the locus coeruleus (LC), which project their axons to regions throughout the brain [1]. LC activity depends on the physical state and behavioral context, and NA modulates various brain functions such as arousal [2], attention, working memory [3], and sensory information processing [4, 5]. For example, in the primary visual cortex (V1), previous electrophysiological studies revealed that iontophoretic administration of NA or electrical LC stimulation modulates neuronal activities [6–12]. Specifically, NA has been reported to enhance the cortical signal-to-noise (S/N) ratio by reducing the spontaneous activity of neurons [6, 8–10] or by enhancing visual responses [12]. These reports suggest that when released to the visual cortex, NA improves behavioral visual detectability, although this hypothesis has not been verified in behaving animals. Moreover, since flashes of light [9, 10] or high contrast flash bars [12] were used as the visual stimuli, the degree to which NA changes the visual detection threshold and whether this effect depends on stimulus spatial characteristics are unknown.

NA has been reported to exert modulatory effects in a receptor-type-specific manner. For example, spontaneous discharges of rat V1 neurons were reduced by NA administered iontophoretically, and the effect was blocked by the co-administration of idazoxan (IDA), an antagonist of α_2_-adrenergic receptors, but not by propranolol (PRP), an inhibitor of β-adrenergic receptors [10]. On the other hand, LC stimulation also reduced spontaneous discharges of rat V1 neurons, but in this case the modulatory effect was blocked by practrol, an antagonist of β-adrenergic receptors, but not by piperoxane or WB4101, two α-adrenergic receptor blockers [7]. Thus, reports have been inconsistent about the effects of inhibitors for α- and β-adrenergic receptors on spontaneous discharges. In addition to those adrenergic receptors, blocking α_1_-adrenergic receptors has been reported to decrease visual responses in the neurons of cat V1 [12], suggesting an enhancement effect of visual responses. Although α- and β-adrenergic receptors are expected to improve visual detectability in animals by enhancing the S/N ratio, this point remains unconfirmed.

Therefore, we focused on three questions, 1) whether NA improves detectability of a behavior-relevant visual stimulus, 2) which receptor subtypes contribute to the NA effects, and 3) whether the NA effects are specific for visual features such as spatial frequency (SF).

To examine the questions, we measured the contrast sensitivity (CS) of freely moving rats as visual detectability of sinusoidal grating stimuli using the two-alternative forced choice (2AFC) visual detection task.

## Methods

### Ethical approval

All experimental protocols were approved by the Research Ethics Committee of Osaka University. All procedures were carried out in compliance with the policies and regulations of the guidelines approved by the Animal Care Committee of the Osaka University Medical School and National Institutes of Health guidelines for the care of experimental animals.

### Animals

Thirteen male Long-Evans rats (200–350 g; Institute for Animal Reproduction, Ibaraki, Japan) were housed one or two per cage (25×40 cm, 20 cm high) on a 12 h light–dark cycle, and the training and task were performed during the light period. The rats in each cage were distinguished by color markers on the tail. The cage was made of plastic and the floor was covered with ammonia-absorbing chips to ease the stench and to keep the animals’ living environment clean and sanitary.

Rats were allowed ad libitum access to pellets every day, but water only on weekends. During the rest of the week, they obtained water by performing the required task during the training and test periods. If the training and test periods exceeded two weeks, free water access was temporarily allowed. We monitored signs of possible dehydration (reduced skin tension and sunken eyes, etc.) twice a day, but none were observed. To ensure adequate hydration, we weighed each animal at the beginning and end of the experiment and compared the weights to a standard weight updated weekly. The weight measured after the session was never <90% standard weight. The institutional animal care and use committee specifically approved the water deprivation protocol.

A veterinarian was available to conduct medical treatment on the ill animals, and a humane endpoint was established as at least one clinical sign of severe illness such as impaired ambulation that prevented the animal from reaching food or water, excessive weight loss and emaciation, lack of physical or mental alertness, difficult or labored breathing, or an inability to remain upright [13]. If animals were judged as humane endpoints, the overdose of urethane would be used for euthanasia. However, no such case occurred in this study. All rats were engaged in this study for maximally 2 months and subsequently kept under normal environmental conditions without sacrifice for future electrophysiological study.

### Apparatus in the 2AFC grating detection task

The behavioral task box was made as described previously [14–16] and is now commercially available from Narishige (EDMS13–264; Tokyo, Japan). A liquid crystal display (LCD) monitor (mean luminance: 30 cd/m^2^) was set at the front side of the box. The box was divided into three areas by translucent walls, and a task-related lever was located at the front center of each area. The lever placed in the middle area was used to begin the trial, and the spout-levers in the other areas were used to deliver water as the reward. Speakers attached to the monitor gave signals indicating trial initiation and auditory feedback of a trial error (200–500 Hz). Rats were monitored through a webcam. Software for the experimental control and stimulus presentation was written in MATLAB (Mathworks, Natick, MA) with extensions from the Psychophysics Toolbox [17, 18].

### Method for teaching the 2AFC grating detection task

Rats were trained in three stages as previously described in detail elsewhere [15]. In the first stage, rats learned to obtain the reward by pulling up a choice lever. In the second stage, the rats learned how to initiate a trial and how to obtain water by detecting a bright patch and executing the basic procedure of the 2AFC task. In the third stage, the grating patch detection training stage, rats learned that the fluid supply was associated with the grating patch.

### Measurement of contrast sensitivity and reaction time

The 2AFC task was combined with a staircase method for direct measurement of the CS. At first, rats pulled up the central lever to start the trial. Then, a circular patch of sinusoidal grating was presented on the right or left side of the monitor. The visual stimulus presentation continued until the rat pulled up a choice lever. The stimulus presentation side was randomly changed trial to trial. The stimulus contrast was set initially at 100%, and it varied from trial to trial according to the animal’s choice (1-up/1-down). Rats were rewarded with water for a correct choice, but then the grating contrast was decreased 1 step in the next trial. After an incorrect choice, only auditory feedback was given, and the stimulus contrast was increased in the next trial. The stimulus contrast was decreased or increased at 1%, 4%, and 10% contrast steps in the low, middle, and high contrast range, respectively. Each session finished when the correct performance in the latest 10 trials fell below 60%. Threshold contrast (C_threshold_) as a percentage was defined as the final stimulus contrast at which the rat was able to choose correctly. CS was calculated as CS = 100/C_threshold_ [19]. C_threshold_ was measured at three SF values (0.1, 0.5, and 1 cycles per degree (cpd)), which were chosen as optimal SF (most contrast-sensitive SF) and two upper limit SFs (lowest contrast-sensitive SF), respectively, based on our previous results from a SF-response function [14].

NA blockers can affect performance in the 2AFC task by modulating behavior other than visual ability including wakefulness or attention. To examine this point, we defined the reaction time of a rat as the time from the central lever manipulation to the choice lever selection.

### Open field test

Locomotor activity was measured by an open field test. The test field was a 60 cm diameter circle divided into nine areas. The activity level was assessed as the number of crossovers between areas for 5 minutes in a dark room. Rat behavior was video-recorded using a web camera and analyzed offline by an observer blind to the experimental conditions.

### Measurement of water intake

The motivation for drinking water was assessed as water intake for 5 minutes in the home cage. Because rats had *ad libitum* access to water from a regular bottle, the water intake was measured as the loss of bottle weight.

### Drugs

Prazosin hydrochloride (PRZ, α_1_-adrenergic receptor antagonist dissolved in 0.9% saline; 1 mg/kg i.p.; Sigma Aldrich, MO), idazoxan hydrochloride (α_2_-adrenergic receptor antagonist dissolved in 0.9% saline; 2 mg/kg i.p.; Sigma Aldrich, MO), propranolol hydrochloride (β-adrenergic receptor antagonist dissolved in 0.9% saline; 10 mg/kg i.p.; Sigma-Aldrich, MO), or 0.9% saline as control condition were injected intraperitoneally 30 min before the 2AFC, open field test and water intake measurement. Drugs doses were those used in previous studies [20–22]. IDA and PRP were tested for seven rats, and PRZ for six rats.

## Results

The α-adrenergic receptor subtypes have been reported to modulate neuronal activity of visual cortex. For example, α_1_ receptor enhanced visual response [11] and α_2_ increased the neuronal S/N ratio of the visual response [9, 10]. This fact led us to expect that intrinsically released NA would improve behavioral CS via α-adrenergic receptors.

**Fig 1:**
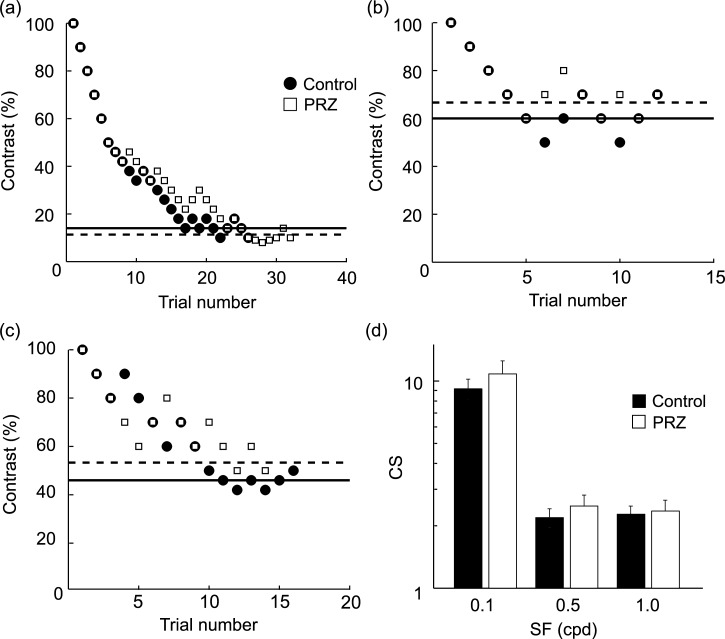
Effects on performance in the 2AFC task by PRZ. (a)-(c) Typical data obtained at three different SFs: 0.1 (a), 0.5 (b), and 1.0 (c) from two sessions of the 2AFC detection task for one rat (black circles, Control; white squares, PRZ). Solid and dotted horizontal lines indicate C_threshold_ of the control and PRZ conditions, respectively. (d) Population data (n = 6) of CS plotted on a logarithmic scale. There was no significant difference between the two conditions at any SF (SF 0.1: *P* = 0.44, SF 0.5: *P* = 0.33, SF 1.0: *P* = 0.58, paired t-test). Error bars are SEM.

To investigate this possibility, we measured CS in freely moving rats using the 2AFC-grating detection task and tested the effects of blocking α_1_-adrenergic receptors by intraperitoneal injection of PRZ at three SFs. Fig 1A–1C shows a typical result of C_threshold_ at different SF where the C_threshold_ was lower at optimal SF (0.1 cpd) than two limited SFs (0.5 and 1 cpd) under no drug conditions in accord with our previous results [14]. Contrary to our expectation, PRZ did not affect C_threshold_ at any SF. The C_threshold_ of the control and PRZ conditions at optimal SF were 14% and 11.3%, respectively (Fig 1A), and the calculated CS was not significantly different between the two drug conditions (Fig 1D; *P* = 0.44, paired t-test). Compared with optimal SF, the limited SF condition raised C_threshold_ and lowed CS under control (no drug) condition (Fig 1B and 1C). No significant difference was observed between the control and PRZ conditions at any SF (Fig 1D; SF 0.5: *P* = 0.30, SF 1.0: *P* = 0.58, paired t-test).

**Fig 2:**
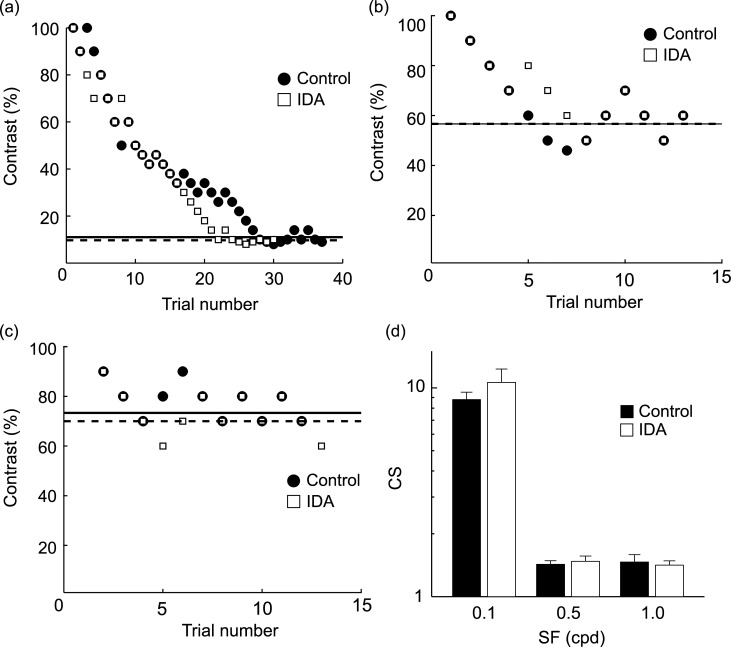
Effects on performance in the 2AFC task by IDA. The format of this figure is identical to that of Fig 1. (a)-(c) Black circles, Control; white squares, IDA. (d) Population data (n = 7) of CS obtained at three different SFs. There was no significant difference between the two conditions at any SF (SF 0.1: *P* = 0.21, SF 0.5: *P* = 0.52, SF 1.0: *P* = 0.47, paired t-test). Error bars are SEM.

Next, we examined the effects of α_2_-adrenergic receptor antagonism by the intraperitoneal injection of IDA at the same three SFs (Fig 2). IDA did not affect C_threshold_ (SF 0.1: Control 11.0%, IDA 9.7%; SF 0.5: Control 56.7%, IDA 56.7%; SF 1.0: Control 73.3%, IDA 70.0%). CS was also unchanged between the control and IDA conditions (Fig 2D; SF 0.1: *P* = 0.21, SF 0.5: *P* = 0.52, SF 1.0: *P* = 0.47, paired t-test).

**Fig 3:**
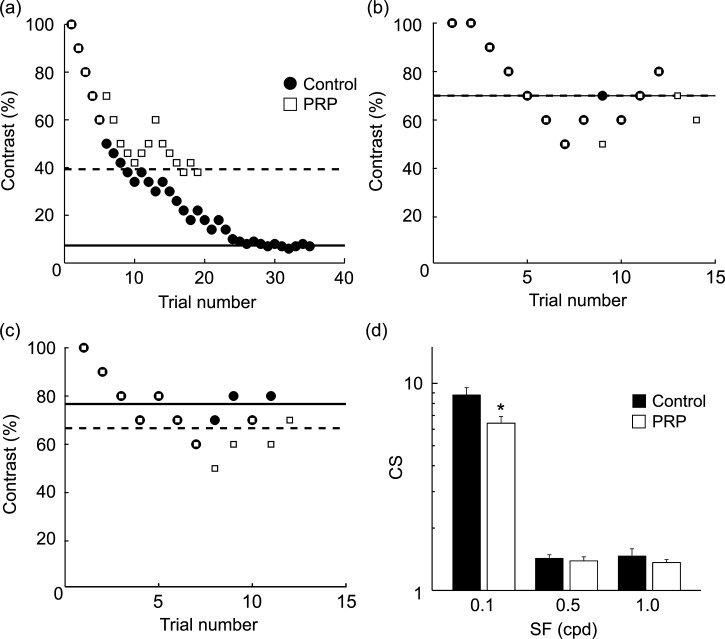
Effects on performance in 2AFC task by PRP. The format of this figure is identical to that of Fig 1. (a)-(c) Black circles, Control; white squares, PRP. C_threshold_ at optimal SF (a) was 7.3% for control (Solid horizontal line) and 39.3% for PRP (dotted horizontal line). (d) Population data (n = 7) of CS significantly decreased in the PRP condition at optimal SF (*P<0.05, paired t-test) but not at limited SFs (SF 0.5: *P* = 0.85, SF 1.0: *P* = 0.47, paired t-test). Error bars are SEM.

A previous study demonstrated that electrical stimulation of the LC attenuates spontaneous discharges of neurons in rat V1 and that this modulatory effect is inhibited by the blockade of β-adrenergic receptors [7]. Therefore, we examined the effects of PRP. PRP raised C_threshold_ at optimal SF (Fig 3A) and significantly decreased CS (Fig 3D; *P* = 0.011, pairs t-test), suggesting that intrinsically released NA contributes to visual detectability via β-adrenergic receptors. However, at limited SF, C_threshold_ was only slightly changed (Fig 3B and 3C) and the resultant CS was unaffected (Fig 3D; SF 0.5: *P* = 0.85, SF 1.0: *P* = 0.47, paired t-test). These results suggest the PRP-induced reduction of CS was dependent on the SF of the grating stimulus. CS at the most sensitive peak SF (0.1 cpd) was significantly attenuated, but not at other SFs (0.5, 1.0 cpd), suggesting that NA improves CS only within a certain SF range.

The blockade of β-adrenergic receptors may lower the motivation to drink water. To examine this possibility, we measured the amount of water drinking, but found no significant difference between drug and control conditions (PRP: 12.2 g; Control: 13.1 g). Since NA is known to change an animal’s spontaneous physical activity level, we also considered it a possible factor in task performance. Therefore, we measured locomotor activity, but found PRP had no effect on it (PRP: 39.2; Control: 45.4). Finally, antagonizing the effect of β-adrenergic receptors may reduce the arousal level or attentional state. Either effect should lead to a delayed reaction time. No significant difference was observed in reaction time, however, between drug and control conditions (SF 0.1: PRP = 2.5 s, Control = 1.8 s; SF 0.5: PRP = 1.9 s, Control = 1.8 s; SF 1.0: PRP = 2.0 s, Control = 1.7 s).

In addition, the amount of water intake was not changed by blocking α-adrenergic receptors with PRZ (PRZ: 8.7 g, Control: 9.2 g) or IDA (IDA: 14.5 g, Control: 13.1 g). Similarly, locomotor activity level was unaffected by PRZ (PRZ: 58.7, Control: 55.5) or IDA (IDA: 44.0, Control: 45.4). The reaction times measured at different SFs (0.1, 0.5, and 1.0) were also unaffected by PRZ (SF 0.1: PRZ = 2.0 s, Control = 1.9 s; SF 0.5: PRZ = 3.1 s, Control = 1.9 s; SF 1.0: PRZ = 2.5 s, Control = 2.0 s) or IDA (SF 0.1: IDA = 2.0 s, Control = 1.8 s; SF 0.5: IDA = 1.9 s, Control = 1.8 s; SF 1.0: IDA = 1.8 s, Control = 1.7 s).

## Discussion

In the present study, we examined whether NA contributes to behavioral visual detectability in freely moving rats. We found that a β-adrenergic receptor antagonist decreased CS only at optimal SF, suggesting that intrinsically-released NA enhances CS in a SF-specific manner in behaving animals.

LC noradrenergic neurons are activated by visual stimuli in visual discrimination tasks for figure shapes [23, 24, 25], and their activity is correlated with task performance [24]. Therefore, intrinsically released NA owing to the visual stimulus-evoked activation of LC noradrenergic neurons might be responsible for the observed CS improvement in the present study.

How does NA improve the behavioral visual performance? One possibility is a modulatory effect on the visual cortex. In general, NA reduces both spontaneous discharges and visual responses in V1 [6, 8–10]. Several studies have demonstrated that the S/N ratio of the visual response is enhanced owing to a relatively dominant inhibitory effect on spontaneous discharges [8–10]. Additionally, electrical stimulation of the LC has been reported to attenuate spontaneous discharges in V1 [7], suggesting that neuronal activity in V1 is directly modulated by NA released from the axonal terminals of LC noradrenergic neurons. Therefore, one possible neuronal mechanism for the improved performance is the enhancement of the S/N ratio in V1.

The present study demonstrated that the NA-induced improvement of behavioral visual detectability is mediated mainly via β-adrenergic receptors but not α-adrenergic receptors. This finding is consistent with an inhibitory effect of LC stimulation on spontaneous discharges in rat V1 [7]. On the other hand, Kolta et al. demonstrated that the activation of α_2_-receptors, but neither α_1_- nor β-receptors, improves the neuronal S/N ratio in anesthetized rat V1 by reducing spontaneous discharges more strongly than visual responses [9, 10]. Since α_2_ receptors act as inhibitory autoreceptors that function at the axonal terminals, systemic administration of IDA could increase the level of NE release as well as block receptor activation, which may obscure the antagonizing effect of α_2_ receptors. Although an increased NE level could decrease the reaction time or increase physical activity, such behavioral changes were not observed in the present study. Another possible explanation for the different results may be attributed to the different brain states due to the presence/absence of anesthetics and effects on the visual cortex due to the different administration methods (iontophoretic local administration versus intraperitoneal injection). Further study is needed to answer this question.

Since drugs were injected intraperitoneally in this study, some peripheral and central factors other than effects on visual areas of the brain could explain the NA-induced improvement of behavioral visual detectability. For example, pupil size is a peripheral factor known to be regulated by NA. However, this regulation was reported to be mediated by α_2_ receptors, not β receptors [26]. Therefore, the CS enhancement in our study seems not to be due to pupil dilation. As central factors, NA effects on arousal level and attentional state may be responsible for the change in CS, but physical activities closely related to arousal level were not changed by the NA blocker in our study. Moreover, reaction times, which reflect the attentional state, were unaffected by the drug. Therefore, the NA effect on CS is unlikely the result of changes in arousal or attention. Finally, we found evidence that NA modulation on CS occurs in a SF-dependent manner. This observation suggests the main affected site was visual cortical areas whose neurons have SF selectivity for the visual stimulus.

To conclude, we show that NA improves the behavioral visual detectability of rat by modulating specific SF. These results suggest that CS at the optimal SF range is dynamically controlled in a behavioral context-dependent manner to realize efficient and flexible visual information processing.
